# Platelet-to-Lymphocyte Ratio as an Independent Factor Was Associated With the Severity of Ankylosing Spondylitis

**DOI:** 10.3389/fimmu.2021.760214

**Published:** 2021-11-05

**Authors:** Tuo Liang, Jiarui Chen, Guoyong Xu, Zide Zhang, Jiang Xue, Haopeng Zeng, Jie Jiang, Tianyou Chen, Zhaojie Qin, Hao Li, Zhen Ye, Yunfeng Nie, Xinli Zhan, Chong Liu

**Affiliations:** ^1^ Department of Spine and Osteopathy Ward, The First Affiliated Hospital of Guangxi Medical University, Nanning, China; ^2^ Graduate School, Guangxi Medical University, Nanning, China

**Keywords:** AS, PLR, nomogram, diagnosis, severity

## Abstract

The study was aimed to determine the association of the platelet-lymphocyte ratio (PLR) with the disease activity of ankylosing spondylitis (AS). A total of 275 patients, including 180 AS patients and 95 non-AS patients, participated in the study. We assessed a full blood count for each participant. Platelet to monocyte ratio (PMR), monocytes to lymphocyte ratio (MLR), monocyte to neutrophil ratio (MNR), platelet to lymphocyte ratio (PLR), neutrophil to lymphocyte ratio (NLR), and platelet to neutrophil ratio (PNR) were calculated. LASSO and logistic regression analyses were performed to establish the nomogram. Receiver operating characteristic (ROC) analysis was performed to evaluate the clinical value of the nomogram. We constructed a novel nomogram, which incorporated easily accessible clinical characteristics like sex, PLR, WBC, EOS, and ESR for AS diagnosis. The AUC value of this nomogram was 0.806; also, the calibration curves indicated a satisfactory agreement between nomogram prediction and actual probabilities. Furthermore, PLR was positively correlated with the severity of AS. PLR was identified as an independent factor for the diagnosis of AS and was associated with the severity of AS.

## Introduction

AS, a chronic inflammatory autoimmune disease, is diagnosed in millions of people every year globally, and it mainly occurs in young adult males (1). AS mostly involves the sacroiliac joints and the axial skeleton and impairs structure and function (2). Its pathogenesis is still unclear but is associated with the presence of human leukocyte antigen B27 (HLA-B27) in 85% to 95% of cases (3, 4).

AS can be diagnosed clinically and radiographically using the modified New York diagnostic criteria (5). There is no specific diagnostic test. Erythrocyte sedimentation rate (ESR) and other acute-phase reactants are usually tested in AS patients for auxiliary diagnosis. However, ESR, C-reactive protein (CRP), and other acute-phase reactants are not related to the disease activity, and changes in ESR are observed in < 50% of patients (6). AS develops due to an immune system disorder. However, AS is a common kind of autoimmune disease, which influences the proportion of immune cells. Therefore, the identification of the immune status changing associated with AS can improve the diagnosis of AS.

In recent years, routine blood parameters were reported as markers of systemic inflammation associated with the diagnosis and prognosis of numerous malignancies and chronic inflammatory diseases (7, 8). A previous study constructed a novel nomogram for the diagnosis of osteoarticular TB by incorporating MLR, ESR and BMI (9).

White blood cells (WBCs) and their counts change in systemic inflammation, including AS. This study aimed to investigate the differences in the complete blood count parameters between AS patients and non-AS patients. Additionally, we also aimed to investigate the relationship between the complete blood count parameters and the severity of AS. We constructed a novel nomogram, which incorporated easily accessible clinical characteristics like sex, PLR, WBC, eosinophils, and ESR for the diagnosis of AS. Additionally, PLR, was identified as an independent factor for the diagnosis of AS and was associated with the severity of AS.

## Patients And Methods

### Patients

Subjects volunteering for the study had signed informed consent forms. The Ethics Committee of The First Affiliated Hospital of Guangxi Medical University approved this study.

From 2012 to 2021, we consecutively screened out 180 AS patients from the First Affiliated Hospital of Guangxi Medical University according to the modified New York criteria (Evaluation of diagnostic criteria for ankylosing spondylitis. A proposal for modification of the New York criteria) (5). In the First Affiliated Hospital of Guangxi Medical University, from 2012 to 2021, a total of 95 non-AS patients were recruited from all the inpatients diagnosed with lumbar disc herniation or lumbar spinal stenosis.

The clinical parameters were obtained from the hospital information system. Information on age, gender, BMI, ESR, CRP, and complete blood count parameters were obtained from all patients. PMR was calculated by dividing the platelet count by the monocyte count, while MLR was calculated by dividing the monocyte count by the lymphocyte count. MNR was calculated by dividing the monocyte count by the neutrophil count, while PLR was calculated by dividing the platelet count by the lymphocyte count. NLR was calculated by dividing the neutrophil count by the lymphocyte count, while PNR was determined by dividing the platelet count by the neutrophil count. Additionally, we obtained the hip Bath AS Radiation Index (BASRI), the Bath Ankylosing Spondylitis Disease Activity Index (BASDAI), and the sacroiliitis grade were also obtained from all patients.

### Statistical Analysis

Statistical analyses were performed and visualized using GraphPad Prism 8. A student’s t-test was used to compare the means of the continuous variables between two groups and the parametric data of the three groups were compared using the one-way ANOVA test. LASSO regression analysis was performed and visualized using the “lars” package in the R software. We selected the factors with the highest lambda values for further analysis (10). The performance of the factors and nomogram was assessed using the ROC curves (“pROC” package) (11). A two-sided probability value less than 0.05 was considered to be statistically significant for all analyses.

## Results

### Nomogram for AS Diagnosis

**Figure 1:**
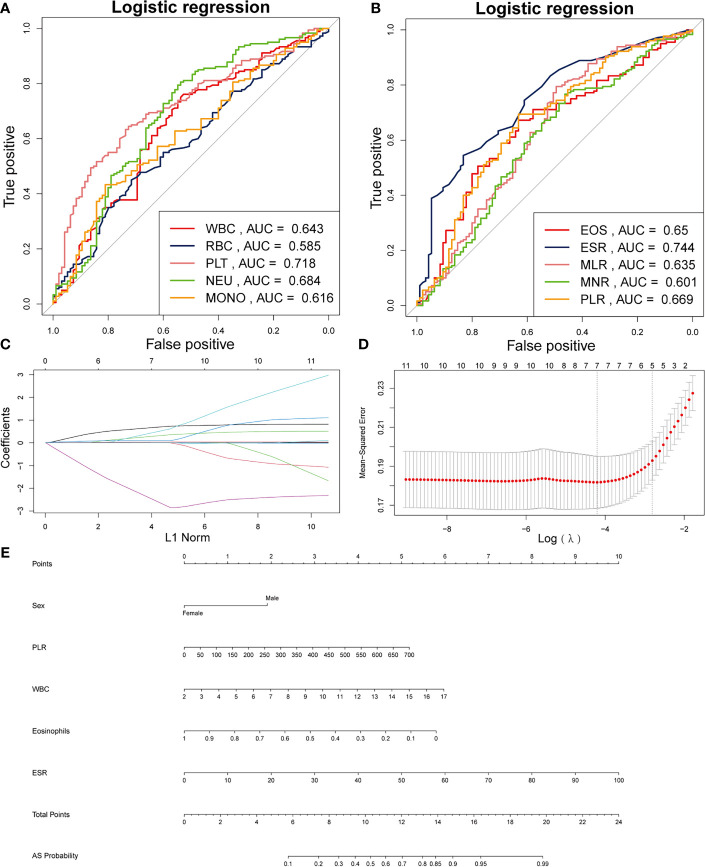
Establishment of a nomogram for AS. **(A)** AUCs of the five factors. **(B)** AUCs of the other five factors. **(C)** Using 1000-fold cross-validation to the optimal penalty parameter lambda. **(D)** LASSO coefficient profiles of the 5 characteristics. **(E)** Nomogram for predicting AS probability.

**Figure 2:**
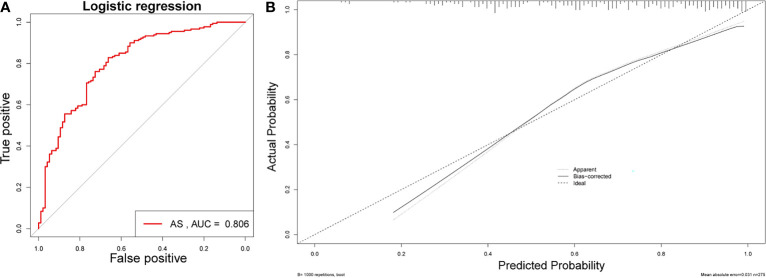
Validation of the nomogram. **(A)** AUC of the nomogram based on the 5 characteristics. **(B)** Calibration curves for predicting AS probability.

**Table 1** illustrates the baseline characteristics collected for the 275 patients, such as age, sex, BMI, and complete blood count parameters. In this study, male patients accounted for 85.6% and constituted the majority of the AS patients. As shown in **Table 1**, WBC, red blood cells (RBC), platelet, neutrophil, monocytes, ESR, MLR, PLR, and NLR were much higher in the AS group than in the non-AS group, while MNR and eosinophils was much higher in the AS group than in the non-AS group. **Figures 1A** **, B** show the value for the area under the curve (AUC) value of the 10 factors that were significantly different between the AS and non-AS groups. A LASSO regression analysis was also performed with the 11 factors to determine the factors to be included in the nomogram model (**Figures 1C** **, D**). Sex, PLR, WBC, eosinophils, and ESR were included in the nomogram (**Figure 1E**). The AUC value of this nomogram was 0.806 (**Figure 2A**); also, the calibration curves indicated a satisfactory agreement between nomogram prediction and actual probabilities (**Figure 2B**).

**Table 1:** Baseline characteristics between AS patients and healthy control.

Characteristics	AS	HC	Overall	P-value
	(N=180)	(N=95)	(N=275)	
**Age**				
Mean (SD)	32.3 (9.07)	35.0 (14.2)	33.2 (11.2)	.086
Median [Min, Max]	30.5 [19.0, 59.0]	36.0 [3.00, 54.0]	32.0 [3.00, 59.0]	
**Sex**				
Female	26.0 (14.4%)	31.0 (32.6%)	57.0 (20.7%)	<.001
Male	154 (85.6%)	64.0 (67.4%)	218 (79.3%)	
**BMI**				
Mean (SD)	22.3 (3.44)	21.6 (4.20)	22.0 (3.73)	.159
Median [Min, Max]	21.6 [9.77, 35.4]	21.2 [13.2, 33.7]	21.5 [9.77, 35.4]	
**WBC**				
Mean (SD)	8.47 (2.12)	7.53 (2.27)	8.15 (2.22)	<.001
Median [Min, Max]	8.18 [3.25, 16.4]	7.10 [2.55, 16.2]	7.91 [2.55, 16.4]	
**RBC**				
Mean (SD)	5.00 (0.677)	4.81 (0.561)	4.93 (0.645)	.013
Median [Min, Max]	4.95 [3.18, 7.41]	4.79 [3.61, 6.70]	4.86 [3.18, 7.41]	
**Platelet**				
Mean (SD)	324 (91.4)	262 (68.0)	302 (89.0)	<.001
Median [Min, Max]	314 [156, 667]	252 [133, 562]	291 [133, 667]	
**Neutrophil**				
Mean (SD)	5.48 (1.84)	4.39 (1.82)	5.10 (1.90)	<.001
Median [Min, Max]	5.33 [1.74, 12.9]	3.97 [1.16, 9.48]	4.90 [1.16, 12.9]	
**Lymphocyte**				
Mean (SD)	2.13 (0.784)	2.24 (0.991)	2.17 (0.861)	.336
Median [Min, Max]	2.08 [0.490, 8.09]	2.04 [0.599, 7.21]	2.06 [0.490, 8.09]	
**Monocytes**				
Mean (SD)	0.682 (0.242)	0.592 (0.216)	0.651 (0.237)	0.002
Median [Min, Max]	0.630 [0.250, 1.81]	0.557 [0.211, 1.33]	0.600 [0.211, 1.81]	
**Eosinophils**				
Mean (SD)	0.193 (0.177)	0.277 (0.204)	0.222 (0.191)	<.001
Median [Min, Max]	0.130 [0, 0.940]	0.243 [0, 0.976]	0.173 [0, 0.976]	
**Basophils**				
Mean (SD)	0.0399 (0.0256)	0.0384 (0.0216)	0.0394 (0.0242)	.596
Median [Min, Max]	0.0400 [0, 0.140]	0.0345 [0, 0.162]	0.0357 [0, 0.162]	
**ESR**				
Mean (SD)	31.1 (20.7)	16.1 (15.1)	25.9 (20.3)	<.001
Median [Min, Max]	27.0 [2.00, 97.0]	11.0 [1.00, 92.0]	20.0 [1.00, 97.0]	
**PMR**				
Mean (SD)	521 (203)	491 (208)	511 (205)	.255
Median [Min, Max]	472 [129, 1420]	468 [181, 1680]	471 [129, 1680]	
**MLR**				
Mean (SD)	0.342 (0.141)	0.297 (0.141)	0.327 (0.143)	.011
Median [Min, Max]	0.309 [0.0980, 1.22]	0.249 [0.0857, 0.839]	0.294 [0.0857, 1.22]	
**MNR**				
Mean (SD)	0.134 (0.065)	0.150 (0.058)	0.140 (0.063)	.048
Median [Min, Max]	0.122 [0.040, 0.59]	0.139 [0.031, 0.30]	0.128 [0.031, 0.59]	
**PLR**				
Mean (SD)	169 (79.9)	136 (69.2)	158 (77.8)	<.001
Median [Min, Max]	154 [46.1, 653]	120 [52.0, 427]	142 [46.1, 653]	
**PNR**				
Mean (SD)	64.1 (23.4)	70.1 (35.8)	66.2 (28.4)	.138
Median [Min, Max]	60.5 [19.7, 182]	65.3 [19.8, 233]	61.2 [19.7, 233]	
**NLR**				
Mean (SD)	2.89 (1.75)	2.32 (1.53)	2.69 (1.70)	.006
Median [Min, Max]	2.53 [0.926, 14.4]	1.88 [0.460, 8.16]	2.34 [0.460, 14.4]	

The red text means that the p value was < 0.05.

### PLR Is Associated With the Severity of AS

**Figure 3:**
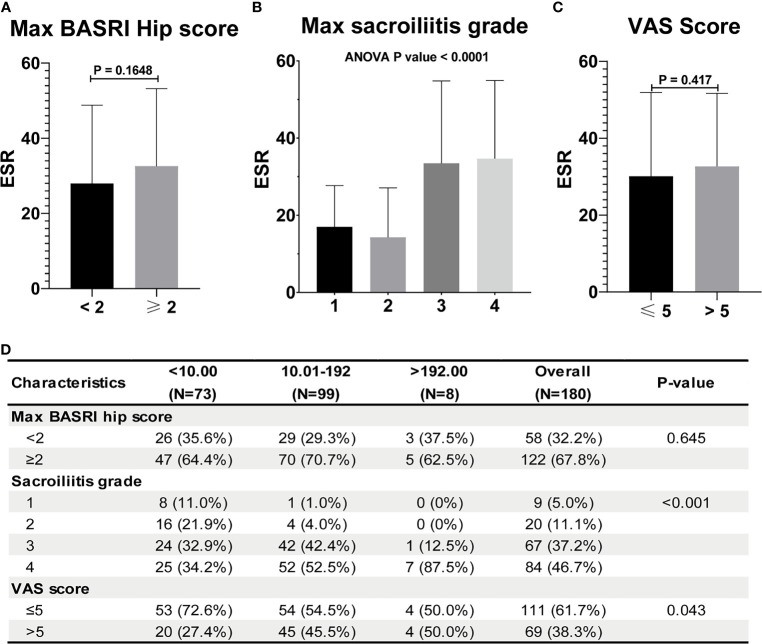
ESR and CRP are not associated with the severity of AS. **(A)** ESR between AS patients with max hip BASRI socre < 2 and ≥ 2. **(B)** ESR in AS patients with different max sacroiliitis grade. **(C)** ESR between AS patients with different VAS pain score. **(D)** Relationship between CRP and the severity of AS.

**Figure 4:**
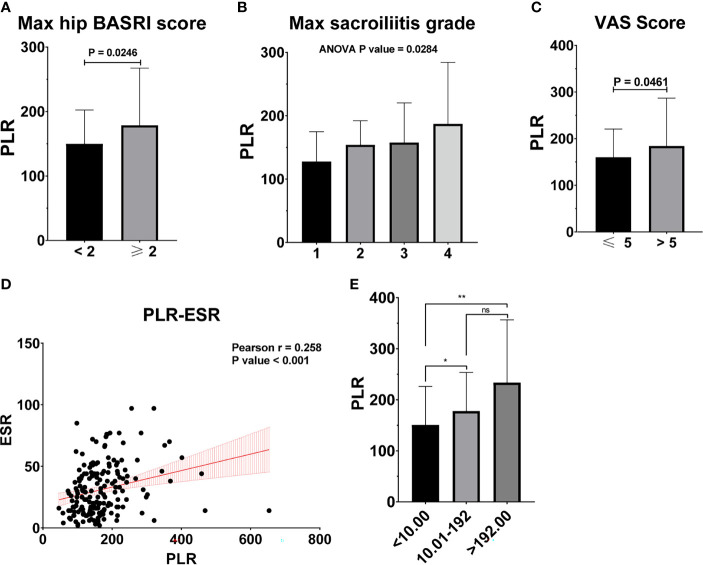
PLR is associated with the severity of AS. **(A)** PLR between AS patients with max hip BASRI socre < 2 and ≥ 2. **(B)** PLR in AS patients with different max sacroiliitis grade. **(C)** PLR between AS patients with different VAS pain score. **(D)** The correlation of PLR with ESR. **(E)** PLR between AS patients with different CRP group. * representative p value < 0.05; ** representative p value < 0.01. ns representative p value > 0.05.

**Figure 5:**
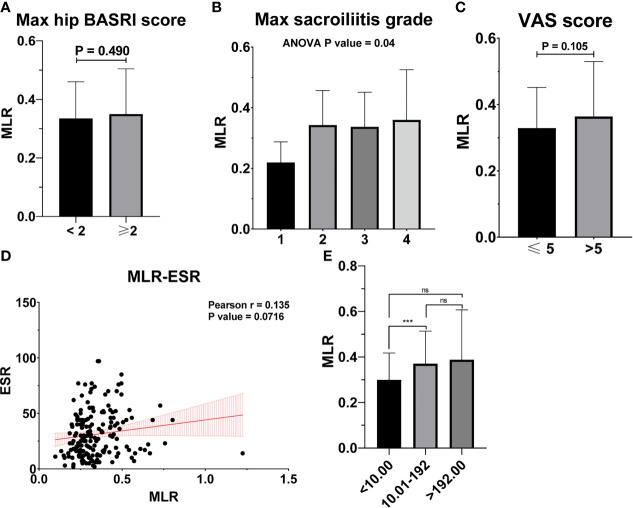
MLR is not associated with the severity of AS. **(A)** MLR between AS patients with max hip BASRI socre < 2 and ≥ 2. **(B)** MLR in AS patients with different max sacroiliitis grade. **(C)** MLR between AS patients with different VAS pain score. **(D)** The correlation of MLR with ESR. **(E)** MLR between AS patients with different CRP group. *** representative p value < 0.001; ns representative p value > 0.05.

**Table 2** shows the baseline characteristics between patients with a BASDAI socre < 4 and those with a BASDAI score ≥ 4. Age, BMI, PLR, and MLR were significantly different between the two groups (**Table 2**). ESR was not associated with the severity of AS (**Figure 3**). Patients with higher CRP had a higher sacroiliitis grade and suffered greater pain, but CRP was not associated with the hip BASRI socre (**Figure 3D**). PLR was significantly higher in AS patients with a max hip BASRI socre ≥ 2 (**Figure 4A**). Furthermore, **Figure 4B** shows that with the increase in the max sacroiliitis grade, the level of PLR also increased. Additionally, patients with higher PLR suffered greater pain (**Figure 4C**). PLR was also positively correlated with ESR (**Figure 4D**). Moreover, PLR was significantly higher in the high CRP group (**Figure 4E**). MLR was not associated with the severity of AS (**Figure 5**).

**Table 2:** Baseline characteristics between BASDAI socre < 4 or ≥ 4 in AS patients.

Characteristics	< 4	≥ 4	Overall	P-value
	(N=152)	(N=28)	(N=180)	
**Age**				
Mean (SD)	31.7 (8.96)	35.5 (9.13)	32.3 (9.07)	0.041
Median [Min, Max]	30.0 [19.0, 59.0]	35.0 [23.0, 59.0]	30.5 [19.0, 59.0]	
**Sex**				
Female	23 (15.1%)	3 (10.7%)	26 (14.4%)	0.75
Male	129 (84.9%)	25 (89.3%)	154 (85.6%)	
**BMI**				
Mean (SD)	22.0 (3.20)	23.7 (4.30)	22.3 (3.44)	0.017
Median [Min, Max]	21.5 [9.77, 35.4]	22.9 [16.2, 35.2]	21.6 [9.77, 35.4]	
**WBC**				
Mean (SD)	8.44 (2.11)	8.68 (2.23)	8.47 (2.12)	0.583
Median [Min, Max]	8.15 [3.25, 16.4]	8.91 [4.40, 14.4]	8.18 [3.25, 16.4]	
**RBC**				
Mean (SD)	5.00 (0.683)	5.00 (0.655)	5.00 (0.677)	0.984
Median [Min, Max]	4.93 [3.49, 7.41]	5.04 [3.18, 6.66]	4.95 [3.18, 7.41]	
**NEU**				
Mean (SD)	5.44 (1.77)	5.67 (2.18)	5.48 (1.84)	0.537
Median [Min, Max]	5.26 [1.74, 12.1]	5.66 [2.78, 12.9]	5.33 [1.74, 12.9]	
**LYM**				
Mean (SD)	2.15 (0.793)	2.00 (0.728)	2.13 (0.784)	0.332
Median [Min, Max]	2.09 [0.490, 8.09]	1.94 [0.90, 4.14]	2.08 [0.490, 8.09]	
**MONO**				
Mean (SD)	0.673 (0.238)	0.727 (0.266)	0.682 (0.242)	0.283
Median [Min, Max]	0.615 [0.25, 1.81]	0.685 [0.34,1.47]	0.630 [0.25, 1.81]	
**EOS**				
Mean (SD)	0.186 (0.169)	0.229 (0.216)	0.193 (0.177)	0.244
Median [Min, Max]	0.135 [0, 0.94]	0.125 [0.008,0.86]	0.130 [0, 0.940]	
**BASO**				
Mean (SD)	0.0388 (0.0257)	0.0458 (0.0245)	0.0399 (0.0256)	0.187
Median [Min, Max]	0.030 [0, 0.140]	0.04 [0.01, 0.130]	0.040 [0, 0.140]	
**PLT**				
Mean (SD)	321 (88.6)	340 (106)	324 (91.4)	0.313
Median [Min, Max]	309 [156, 667]	334 [189, 588]	314 [156, 667]	
**ESR**				
Mean (SD)	30.1 (21.2)	36.7 (17.3)	31.1 (20.7)	0.122
Median [Min, Max]	25.0 [2.00, 97.0]	37.0 [5.00, 77.0]	27.0 [2.00, 97.0]	
**MLR**				
Mean (SD)	0.333 (0.136)	0.393 (0.158)	0.342 (0.141)	0.04
Median [Min, Max]	0.306 [0.0980, 1.22]	0.320 [0.220, 0.758]	0.309 [0.0980, 1.22]	
**MNR**				
Mean (SD)	0.133 (0.0661)	0.140 (0.0588)	0.134 (0.0649)	0.605
Median [Min, Max]	0.121[0.05,0.592]	0.122[0.04,0.318]	0.122 [0.04,0.592]	
**PMR**				
Mean (SD)	524 (205)	509 (197)	521 (203)	0.727
Median [Min, Max]	475 [198, 1420]	469 [129, 1130]	472 [129, 1420]	
**PLR**				
Mean (SD)	164 (69.6)	197 (119)	169 (79.9)	0.045
Median [Min, Max]	154 [46.1, 468]	152 [84.4, 653]	154 [46.1, 653]	
**PNR**				
Mean (SD)	63.8 (23.1)	65.3 (25.6)	64.1 (23.4)	0.756
Median [Min, Max]	61.2 [19.7, 182]	58.1 [23.8, 131]	60.5 [19.7, 182]	
**NLR**				
Mean (SD)	2.79 (1.48)	3.41 (2.77)	2.89 (1.75)	0.086
Median [Min, Max]	2.51 [0.926, 13.8]	2.60 [0.97, 14.4]	2.53 [0.926, 14.4]	

The red text means that the p value was < 0.05.

### Nomogram Predictions for Activate Patient With AS

**Figure 6:**
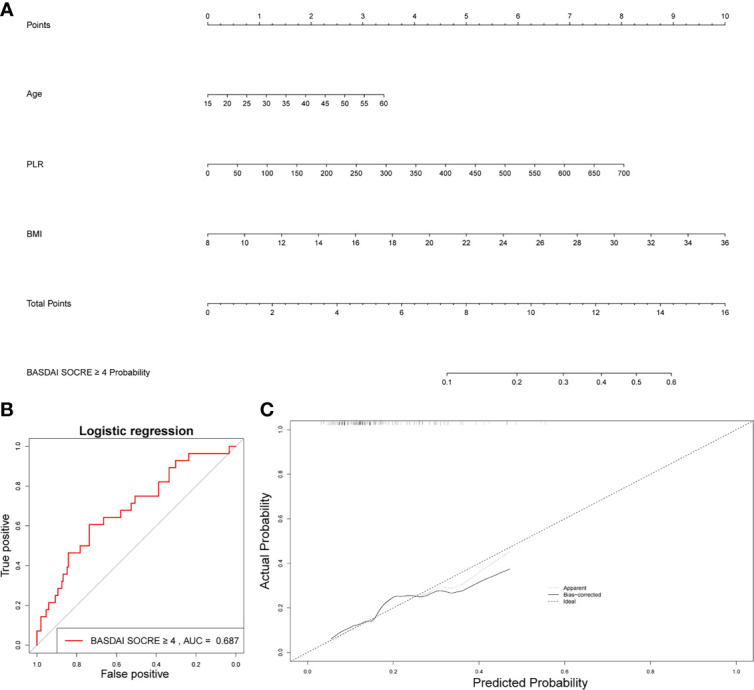
Establishment of a nomogram for activated AS patient prediction. **(A)** The nomogram for activated AS patient prediction. **(B)** AUC of the nomogram based on the 3 characteristics. **(C)** Calibration curves for predicting activated AS patient prediction.

Age, BMI, and PLR were included in the prediction nomogram for activated AS patient prediction (**Figure 6A**). Moreover, the ROC analysis showed that the AUC value of the nomogram was 0.686 (**Figure 6B**). The calibration curves of this nomogram are shown in **Figure 6C**.

## Discussion

Platelets interact with many types of cells, including immune cells and stromal cells. Interestingly, previous studies showed that platelets also interact with osteoblasts (12, 13). Platelet-rich plasma (PRP) and platelet-rich fibrin were reported to be positively correlated with osteoblast differentiation, proliferation, and migration (14). Quiescent or very slowly dividing osteoblasts showed a burst of proliferation after platelet stimulation and returned to a non-dividing or very slowly dividing condition when platelets were removed. Besides, freeze-dried PRP induced osteoblast proliferation *via* platelet-derived growth factor receptor-mediated signal transduction (15). These studies demonstrated that platelet were strongly associated with bone formation. In this study, platelet levels were significantly increased in AS patients, which suggested that high levels of platelets were associated with heterotopic ossification.

Platelets play a central role in primary hemostasis, adhering to the damaged vascular bed caused by subendothelial collagen exposure (16). Another important (though less commonly evoked) function of platelets is their active participation in anti-infectious responses (17). Additionally, platelets orchestrate the immune response by modulating several immune cells (18, 19). Presently, HLA-B27, acute-phase reactants, clinical characteristics, and radiographically detected changes in images are the main ways to diagnose AS. Many patients are HLA-B27 (–) whose early clinical and imaging results are not atypical, which poses great diagnostic challenges to us.

Previous studies demonstrated that the complete blood count parameters and the ratio between them might act as markers for prognosis, status, and progression of diseases (20, 21). PLR was reported to be closely related to the progression and prognosis of acute ischemic stroke, knee osteoarthritis, and papilledema due to idiopathic intracranial hypertension (22, 23). AS is a chronic inflammatory autoimmune disease, and we aimed to determine the relationship between AS and PLR. Similar to the systemic inflammatory indices related to the immune system, PLR can be directly obtained from the whole blood cell count, which is convenient and cheap. The results of this study demonstrated that the levels of PLR were significantly higher in the AS group than in the non-AS group. PLR was also an independent factor for the diagnosis of AS. By including in sex, PLR, WBC, eosinophils, and ESR, we constructed a nomogram, which could distinguish AS patients from patients with low back pain.

Moreover, PLR can also be used as an indicator of disease severity in AS patients. Elevated levels of CRP or ESR have been found in only about 60% of clinically-active AS patients (8). The clinical assessment of disease activity and response to treatment in AS is complex and difficult. Although the two traditional markers of an acute phase response, ESR and CRP, have been used for assessment, they may not often correlate with the patient’s symptoms or radiological progression (8). In the present study, PLR was correlated with multiple clinical characteristics in AS patients. In the BASDAI ≥4 activate AS patient group, PLR significantly increased, compared to the BASDAI < 4 inactivate AS group. The level of PLR increased with the progression of the max sacroiliitis grade. Additionally, PLR was significantly higher in AS patients with a max hip BASRI socre ≥2 compared to the AS patients with a max hip BASRI socre < 2.

The increase in PLR was caused by the increase in the platelet count and the decrease in the lymphocyte count in peripheral blood, as found in other systemic inflammatory reactive diseases. ESR and CRP are one of the most commonly used indicators of the degree of inflammation. However, ESR was not associated with the severity of AS in our study (**Figure 3**). Patients with higher CRP had a higher sacroiliitis grade and suffered greater pain, but CRP was not associated with the hip BASRI socre. In this study, the PLR were positively correlated with ESR and CRP. Patients with an elevated PLR had a higher VAS pain score. PLR, along with its potential to predict disease severity, was investigated in several neoplastic, prothrombotic, and metabolic diseases. Its role has been attributed to increased thrombogenic activity (24–26). These results demonstrated that PLR could be used as an indicator of disease severity in AS patients. These hematological ratios can help to categorize the disease severity and progression in patients, thereby enabling us to make appropriate and informed clinical decisions. To some extent, PLR can make up for the deficiency of ESR and CRP in assessing the severity of AS.

In the BASDAI ≥ 4 activated AS patient group, MLR also increased significantly, compared to the BASDAI < 4 inactivated AS group. But MLR was not correlated with other clinical characteristics such as the hip BASRI socre. MLR could be used as an indicator of disease severity in AS patients. Finally, based on three factors, including age, PLR, and BMI, we constructed a nomogram, which could predict the possibility of BASDAI ≥ 4 in AS patients. Obtaining data on these factors are convenient and affordable.

This study had some limitations. First, this retrospective cohort study recruited only patients from only single institution. Therefore, a certain risk of bias remains. Second, multi-center clinical trials with a larger sample size are still needed.

## Conclusion

PLR, was identified as an independent factor for the diagnosis of AS and was associated with the severity of AS.

## Data Availability Statement

The original contributions presented in the study are included in the article/supplementary material. Further inquiries can be directed to the corresponding authors.

## Ethics Statement

All subjects volunteered for the study and signed informed consent forms. In order to ensure confidentiality, the names of study participants were not included in the data. Information obtained from the data of the study participants is kept confidential. In addition, the Ethics Committee of The First Affiliated Hospital of Guangxi Medical University approved the study.

## Author Contributions

XZ and TL: Conceptualization, Methodology. JC, GX, and ZZ: Data curation, Investigation. JX, HZ, and JJ: Formal analysis, Software. ZQ, HL, TC, YZ, and YN: Visualization. TL and CL: Writing- Reviewing and Editing. All authors contributed to the article and approved the submitted version.

## Funding

This work was sponsored by the National Natural Science Foundation of China (81560359); National Natural Science Foundation of China (81860393). Funding bodies had no role in the study design, collection, analysis, and interpretation of the data or in writing the manuscript.

## Conflict of Interest

The authors declare that the research was conducted in the absence of any commercial or financial relationships that could be construed as a potential conflict of interest.

## Publisher’s Note

All claims expressed in this article are solely those of the authors and do not necessarily represent those of their affiliated organizations, or those of the publisher, the editors and the reviewers. Any product that may be evaluated in this article, or claim that may be made by its manufacturer, is not guaranteed or endorsed by the publisher.

## Abbreviations

AS, ankylosing spondylitis; PMR, platelet to monocyte ratio; MLR, monocytes to lymphocyte ratio; MNR, monocyte to neutrophil ratio; PLR, platelet to lymphocyte ratio; NLR, neutrophil to lymphocyte ratio; PNR, platelet to neutrophil ratio; BASRI, Bath Ankylosing Spondylitis Radiation Index; BASDAI, Bath Ankylosing Spondylitis Disease Activity Index; ESR, erythrocyte sedimentation rate; CRP, C-reactive protein; WBC, whole blood cell; PRP, Platelet-rich plasma; HLA-B27, human leukocyte antigen B27; RBC, red blood cells; AUC, area under the curve; ROC, Receiver operating characteristic; BMI, body mass index.
